# Integrating machine learning, bioinformatics and experimental verification to identify a novel prognostic marker associated with tumor immune microenvironment in head and neck squamous carcinoma

**DOI:** 10.3389/fimmu.2024.1501486

**Published:** 2024-12-10

**Authors:** Xiaoxia Zeng, Dunhui Yang, Jin Zhang, Kang Li, Xijia Wang, Fang Ma, Xianqin Liao, Zhen Wang, Xianhai Zeng, Peng Zhang

**Affiliations:** ^1^ Department of Otolaryngology, Longgang Otolaryngology hospital & Shenzhen Key Laboratory of Otolaryngology, Shenzhen Institute of Otolaryngology, Shenzhen, Guangdong, China; ^2^ Department of Otolaryngology, The Second People’s Hospital of Yibin, Yibin, Sichuan, China

**Keywords:** ITM2A, immune microenvironment, HNSC, machine learning, prognostic

## Abstract

Head and neck squamous carcinoma (HNSC), characterized by a high degree of malignancy, develops in close association with the tumor immune microenvironment (TIME). Therefore, identifying effective targets related to HNSC and TIME is of paramount importance. Here, we employed the ESTIMATE algorithm to compute immune and stromal cell scores for HNSC samples from the TCGA database and identified differentially expressed genes (DEGs) based on these scores. Subsequently, we utilized four machine learning algorithms to identify four key genes: ITM2A, FOXP3, WIPF1, and RSPO1 from DEGs. Through a comprehensive pan-cancer analysis, our study identified aberrant expression of ITM2A across various tumor types, with a significant association with the TIME. Specifically, ITM2A expression was markedly reduced and correlated with poor prognosis in HNSC. Functional enrichment analysis revealed that ITM2A is implicated in multiple immune-related pathways, including immune-infiltrating cells, immune checkpoints, and immunotherapeutic responses. ITM2A expression was observed in various immune cell populations through single-cell analysis. Furthermore, we showed that ITM2A overexpression inhibited the growth of HNSC cells. Our results suggest that ITM2A may be a novel prognostic marker associated with TIME.

## Introduction

Head and neck squamous carcinoma (HNSC) is a neoplasm predominantly originating in the oral cavity, pharynx, larynx, nasal cavity, and salivary glands (1). The etiology of HNSC is multifactorial, with significant associations to smoking, alcohol consumption, human papillomavirus infection, as well as environmental and genetic factors (2). Advances in technology and therapeutic interventions have enhanced the early detection and treatment of HNSC, leading to relatively high five-year survival rates for patients diagnosed at an early stage. However, the survival rate of late-stage patients is still no more than 50% (3). Therefore, it is urgent to develop new treatments and therapeutic targets.

The tumor immune microenvironment (TIME) encompasses the milieu surrounding tumor cells, which includes cancer cells, the extracellular matrix, immune cells, stromal cells and cytokines (4). The TIME represents a highly intricate and dynamic system that exerts a profound influence on tumorigenesis, progression, metastasis, and therapeutic response. In the contexts of classical Hodgkin lymphoma, pancreatic ductal adenocarcinoma, and glioblastoma multiforme, modulation of the TIME has been demonstrated to enhance therapeutic outcomes. In colorectal adenocarcinoma (COAD), researchers have investigated immune-related therapeutic targets within the TIME (5). HNSC treatment was improved by immunomodulation of TIME (6, 7). Therefore, it is important to analyze TIME and related immune-infiltrating cells, which will help to screen new targets for improving HNSC treatment and prognosis.

ITM2A is a protein-coding gene that is part of the ITM2 family of type II membrane proteins (8). In the context of lung cancer, miRNA-143-3p facilitates the proliferation of lung cancer cells by targeting and regulating ITM2B (9). Similarly, in esophageal squamous cell carcinoma, microRNA-196a-5p promotes tumorigenesis and progression through its interaction with ITM2B (10). Furthermore, ITM2C has been identified as a diagnostic biomarker for colorectal cancer and is correlated with the prognosis of breast cancer (11, 12). Similarly, ITM2A has been shown to be a tumor suppressor and is associated with PD-L1 in breast cancer (13). In bladder cancer, ITM2A inhibits bladder cancer by downregulating STAT3 phosphorylation (14). However, it is still unknown about ITM2A in HNSC.

Here, we demonstrated the ITM2A is associated with TIME, prognosis and progression of HNSC by bioinformatics analysis. We also analyzed the relationship between ITM2A and the immune microenvironment, immune cell infiltration, distribution, and immunotherapy. Moreover, the effects of ITM2A on HNSC growth were verified by cell and animal experiments. These findings suggest that ITM2A may be a novel prognostic and immune-related biomarker for HNSC.

## Materials and methods

### Data download

FPKM of HNSC was downloaded from The Cancer Genome Atlas (TCGA). The dataset included information on 522 HNSC cases and 44 normal head and neck tissues.

### Calculation of ImmuneScore, StromalScore, and ESTIMATEScore

TIME analysis was calculated by the ESTIMATES R software package.

### Selection of DGEs

We first grouped the patients based on ImmuneScore, StromalScore, and tumor cases and normal cases. Next, difference analysis was performed by limma R package, and the screening threshold was |logFC|>1, p<0.05. Finally, the VennDiagram R software package was applied to take intersection analysis of DGEs and visualize them.

### Univariate Cox analysis

DGEs associated with patient survival and hazard ratios (HRs) were used to identify risk (HR>1) or protective (HR<1) genes.

### Machine learning algorithm to screen key genes

In order to identify key biomarkers associated with HNSC immune prognosis, we obtained data from HNSC patients from the TCGA database and constructed corresponding gene profiles. We used LASSO algorithm of the glamnet R package, the SVM-RFE algorithm of the e1071 R package, GBM algorithm of the gbm R package and the randomForest R package, respectively, to screen for key biomarkers. The results of the algorithms were finally intersected by the VennDiagram R package to identify the key biomarkers associated with immune prognosis in HNSC (15).

### Analysis of pan-cancer differences

ITM2A mRNA expression was analyzed by the Human Protein Atlas. Then, we compared the ITM2A gene expression from pan-cancer in TCGA through the TIMER database (http://timer.cistrome.org/) (16). To further validate our findings, UCSC XENA database was applied to obtain data from tumor samples in TCGA and the corresponding normal tissue data in GTEx (17, 18).

### Analysis of pan-cancer immune infiltration

ssGSEA algorithm in the GSVA R package was used to calculated immune infiltration (19). For TCGA pan-cancer data, we analyzed the correlation between single-gene expression and immune infiltration using the Spearman method and visualized the results with the ggplot2 R package (20).

### Survival analysis

After excluding some samples with missing data, we evaluated the prognostic impact of ITM2A using the TCGA-HNSC dataset and related clinical information. The TCGA-HNSC samples were categorized into ITM2A-high and ITM2A-low based on the median ITM2A expression. Subsequently, Kaplan-Meier survival curves was used to visualized overall survival (OS).

### ROC analysis

We performed a ROC analysis of ITM2A using the pROC R package on ITM2A expression and clinical information in the TCGA-HNSC cohort. With this analytical approach, we were able to determine the diagnostic efficacy of ITM2A.

### Difference analysis

To analyze the DEGs of samples from the ITM2A high and low expression groups in TCGA-HNSC, we performed a difference analysis using the Limma R software package with a threshold of |logFC|>1, FDR<0.05. A total of 522 HNSC samples and 44 control samples were utilized.

### Functional enrichment

Gene Ontology (GO) and Kyoto Encyclopedia of Genomes (KEGG) enrichment analysis of ITM2A-associated DEGs in the TCGA-HNSC cohort were analyzed by the clusterProfiler R package. Gene Set Enrichment Analysis (GSEA) was performed by the c2.cp.kegg.v7.4.symbols.gmt reference gene set.

### Immune infiltration analysis

Immune-infiltrating cells was analyzed by the CIBERSORT R software package. Additionally, we used the Sperarman method to determine the correlation between ITM2A expression and immune-infiltrating cells (21). Finally, the results were visualized by linkET and ggplot2 R software packages.

### Immunotherapy and immune checkpoint analysis

To investigate the impact of ITM2A on immunotherapy, we downloaded the Immunophenotype Score file (IPS) from the TCIA (https://tcia.at/) database for patients in the TCGA-HNSC cohort to explore whether there was a difference in therapeutic efficacy of PD1 and CTLA inhibitors between subgroups with high and low ITM2A expression. Subsequently, correlation analyses of ITM2A and immune checkpoint genes (ICP) were performed by the Person method. p<0.001 was considered statistically significant and visualized by the ggplot2 R package (22).

### Drug sensitivity analysis

We calculated the IC50 of the ITM2A high expression group (ITM2A-high) and the ITM2A low expression group (ITM2A-low) designated as representative of the drug half-inhibitory efficiencies in a comparative manner, respectively, using the pRRophetic R software package (23).

### Single-cell transcriptome data analysis

We analyzed the GEO dataset (GSE139324) using the Tumor Immune Single-cell Hub (http://tisch.comp-genomics.org/home/, TISCH) database (24). All cells were clustered, annotated and visualized by applying the Uniform Mobility Approximation and Projection (UMAP) method.

### Cell Culture

SCC9 and CAL27 cells were from BeNa Culture Collection. The medium used for the cells was DMEM medium supplemented with 10% FBS.

### ITM2A overexpression

Human ITM2A ORF nucleotide sequence (GenBank NM_004867) was cloned into pCMV6-Entry vector. SCC9 and CAL27 cells were transfected with ITM2A plasmid and empty plasmid vector using Lipofectamine™ 3000 reagent (Invitrogen) according to the manufacturer’s instruction.

### Western blotting

Western blotting experiments were performed as previously described (25). The PVDF membrane was incubated with anti-ITM2A (Proteintech) or β-Actin (Santa) at 4°C overnight. Then incubated it with secondary antibody (Cell Signaling) for 2 hours. Finally, Pierce™ ECL Protein Immunoblotting Substrate (Thermo Scientific) was used to detect the bands.

### Colony formation assay

The cells were seeded into 6-well plates with 600 cells per well and cultured for 14 days. Then we fixed the cells using 4% paraformaldehyde and stained the cells using crystal violet.

### RT-qPCR

We extracted total RNA using the RNeasy Mini kit (Qiagen). Subsequently, RT Master Mix (MedChemExpress) was used to obtain cDNA. Finally, SYBR Green qPCR Master Mix (MedChemExpress) was used to perform qPCR experiments on Applied Biosystems 7500 FAST Real Time PCR system. *ACTB* forward 5′-CACCATTGGCAATGAGCGGTTC-3′, reverse 5′-AGGTCTTTGCGGATGTC CACGT-3′. *ITM2A* forward 5′-GGCAGGACTTATTGTTGGGTGGGAG-3′, reverse 5′-CCTCAGTCACAGGCAGGAAGTT-3′.

### Cell viability assay

The cells were cultured for 0-72 hours. Subsequently, we added 10 μl of CCK-8 reagent (MedChemExpress) to each well, and then incubated the 96-well plate in an incubator at 37°C for 2 hours. We detected the absorbance values at 450 nm using a multi-mode plate reader (Molecular Devices).

### Immunohistochemistry

Immunohistochemistry was performed as previously described (25). The German semi-quantitative scoring system (no staining =0, weak staining = 1, moderate staining = 2, strong staining = 3) and the percentages of stained cells (0% = 0, 1-24% = 1, 25-49% = 2, 50-74% = 3, 75-100% = 4) were used to assess the results. The final immune reactive score was determined by multiplying the intensity score by the percentage score, ranging from 0 to 12.

### Tumor xenograft experiments

4-week old male BALB/c nude mice (n=20) were purchased from Guangdong Medical Laboratory Animal Center. The transfected SCC9 and CAL27 cells (2 x 10^6^) were injected into the dorsal flank of the mice. Tumor volumes were measured every 3 days: volume = (width)² × length/2. Tumor growth was plotted against time. The mice were euthanized by CO_2_, then the tumors were taken out and taken pictures (25).

### Statistical analysis

R and GraphPad Prism were used to statistical analyses. We conducted at least three independent experiments and expressed it as mean ± standard error (SEM). p-values less than 0.05 were considered statistically significant.

## Results

### Screening DEGs based on ImmuneScore and StromalScore

**Figure 1:**
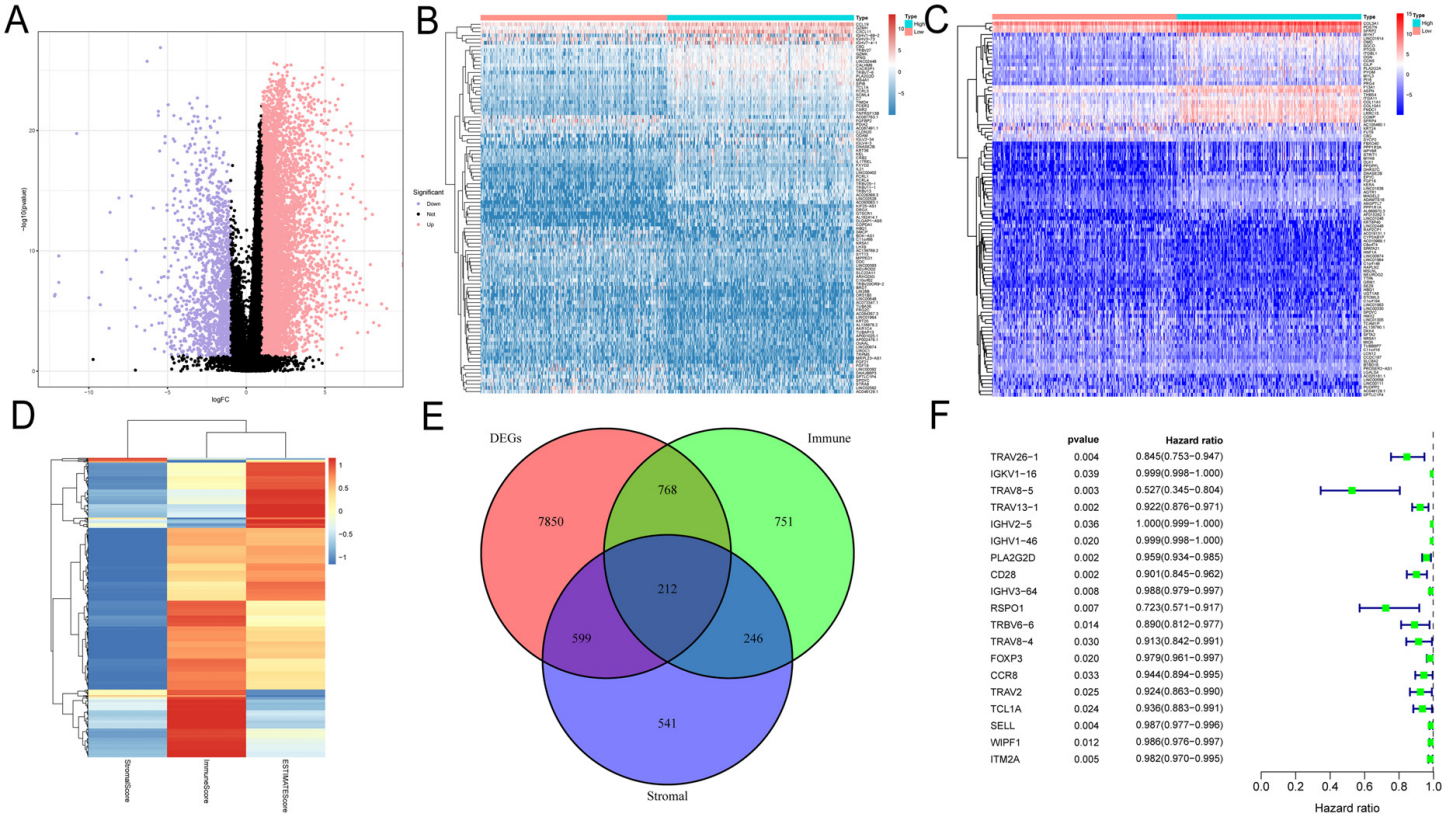
Identification of prognosis-related genes based on ImmuneScore, StromalScore and DEG. **(A)** Volcano plots were used to show the distribution of DEGs. **(B–D)** Heatmaps were used to show DEG expression in ImmuneScore and StromalScore, respectively. **(E)** Wayne plots showing intersecting genes in ImmuneScore, StromalScore and DEG. **(F)** Univariate Cox analysis was used to screen the prognosis-related genes among the intersection genes.

DEGs were identified and demonstrated by volcano plots (**Figure 1A**). Next, we applied the ESTIMATE algorithm to calculate the ImmuneScore, StromalScore, and ESTIMATEScore. Based on this, we screened 1977 and 1598 DEGs from HNSC samples, and the heatmap demonstrated the ranking of the top 50 DEGs (**Figures 1B–D**). To identify DEGs associated with the TIME, we performed intersection screening of these three groups of DEGs using the Venn diagram tool, and identified 212 shared genes (**Figure 1E**). Subsequently, twenty genes were associated with TIME and had a significant prognostic impact (**Figure 1F**).

### Screening TIME-related prognostic genes using multiple machine learning algorithms

**Figure 2:**
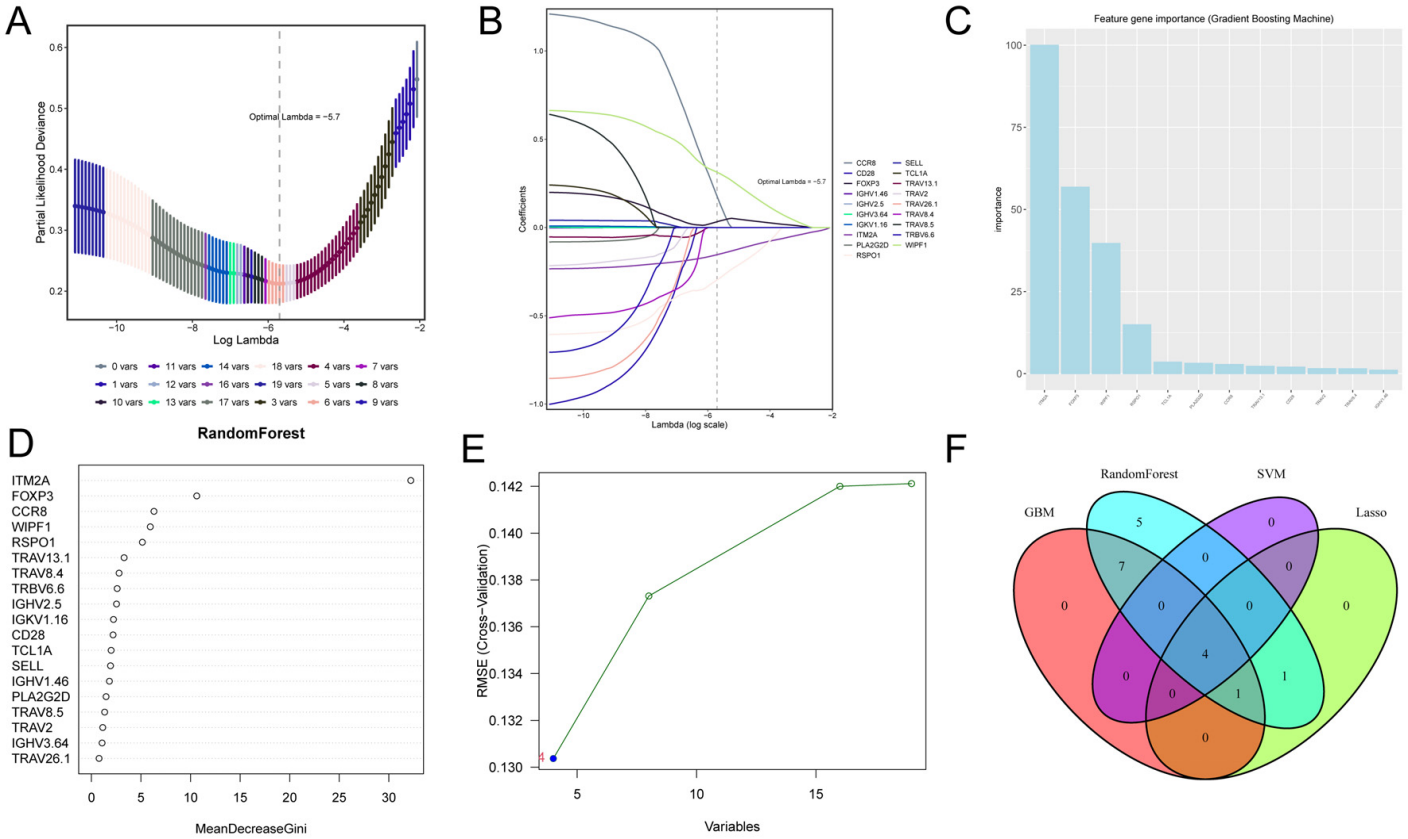
Screening of candidate genes associated with TIME and prognosis in HNSC using four machine learning algorithms. **(A, B)** Shows the optimal parameter lambda value selected in the LASSO model and the variation of different genes with the Lamdba value. **(C)** The top 10 most important genes in the gradient boosting model. **(D)** Top 18 most important genes in the random forest model. **(E)** Optimal values of RMSE in the SVM-RFE algorithm model. **(F)** Wayne plots for filtering important genes for four models, LASSO regression, Support Vector Machine - Recursive Feature Elimination, Random Forest and Gradient Booster.

Different machine learning algorithms each have unique characteristics and advantages, but a single algorithm may produce bias or error. To improve the accuracy and reliability of screening key genes, we used four machine learning algorithms to identify TIME-related prognostic key genes. The cross-validation of the LASSO regression model’s error was minimized when the value of λ was 0.57, which means that the model’s predictive performance was optimal at this point. The final screened key genes included IGHV2.5, RSPO1, FOXP3, CCR8, WIPF1 and ITM2A (**Figures 2A, B**). In the feature importance analysis of the gradient boosting machine (GBM) model, the top five key genes were ITM2A, FOXP3, WIPF1, RSPO1, and TCL1A (**Figure 2C**). The feature importance plot of the random forest model showed that ITM2A, FOXP3 and CCR8 had the highest importance scores in the random forest model (**Figure 2D**). In SVM-RFE model, the root mean square error (RMSE) of the model appeared to change as the number of variables increased. When the number of variables was 4, the RMSE of the model reached the lowest, and the key genes finally screened out included ITM2A, FOXP3, RSPO1 and WIPF1 (**Figure 2E**). Finally, we demonstrated the overlap of the key genes screened by the four machine learning algorithms through Venn diagrams, and the results showed that ITM2A, FOXP3, WIPF1 and RSPO1 were screened in all the models (**Figure 2F**).

### ITM2A is closely related to TIME in pan-cancer

**Figure 3:**
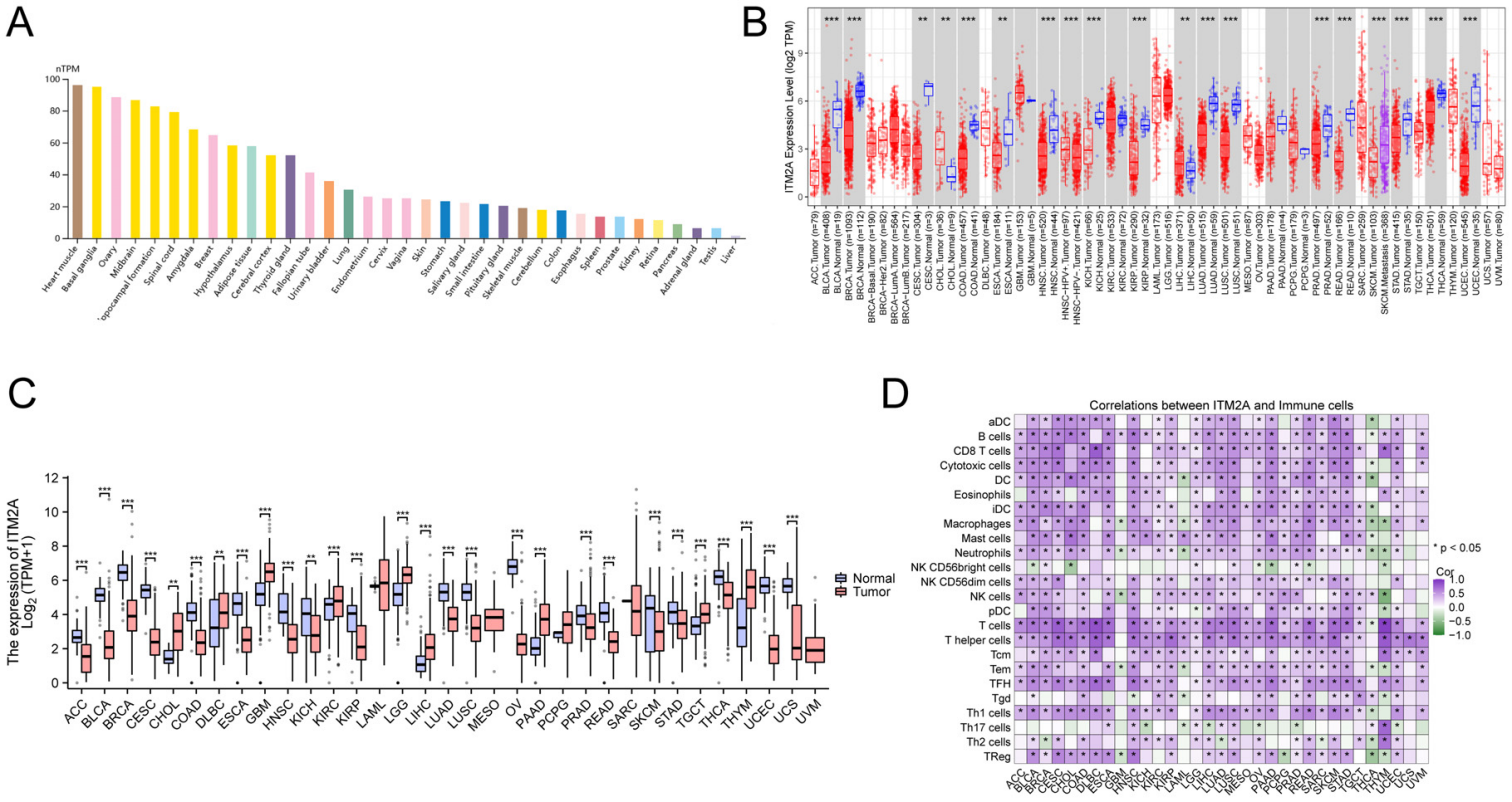
Expression of ITM2A in different tissues and cancer types. **(A)** Expression levels of ITM2A in various normal tissues. **(B)** Expression level of ITM2A in pan-cancer in TCGA database. **(C)** Expression levels of ITM2A in pan-cancer in the TCGA combined GTEx database. **(D)** Heatmap of the correlation between ITM2A in pan-cancer and immune cell infiltration using the ssGSEA algorithm. *p<0.05, **p<0.01, ***p<0.001.

By analyzing the HPA data, we found that the mRNA of ITM2A was widely expressed in normal tissues, such as myocardium, basal ganglia, ovary, and midbrain, prostate, kidney, retina, pancreas, adrenal gland, testis, and liver (**Figure 3A**). DiffExp module of the TIMER database showed that ITM2A showed abnormally lower expression in tumor tissues of BLCA, CESC BRCA, ESCA, COAD, HNSC, KICH, KIRP, STAD, LUAD, THCA, LUSC (**Figure 3B**). To gain a deeper understanding of the difference in ITM2A expression in normal and tumor tissues, we analyzed the TCGA combined with GTEx pan-cancer data using XENA download. The results revealed that ITM2A was significantly reduced in ACC, BLCA, BRCA, CESC, COAD, ESCA, HNSC, KICH, KIRP, LUAD, LUSC, OV, PRAD, READ, SKCM, STAD, THCA, UCEC, and UCS tumors, (**Figure 3C**). Finally, we analyzed the immune cell infiltration by ssGSEA algorithm using TCGA pan-cancer data, and correlation analysis between ITM2A and immune infiltration matrix data was performed using Spearman’s method. The results showed that ITM2A and most immune cell types had a significant correlation in most tumors (**Figure 3D**).

### ITM2A is associated with poor HNSC patient prognosis

**Figure 4:**
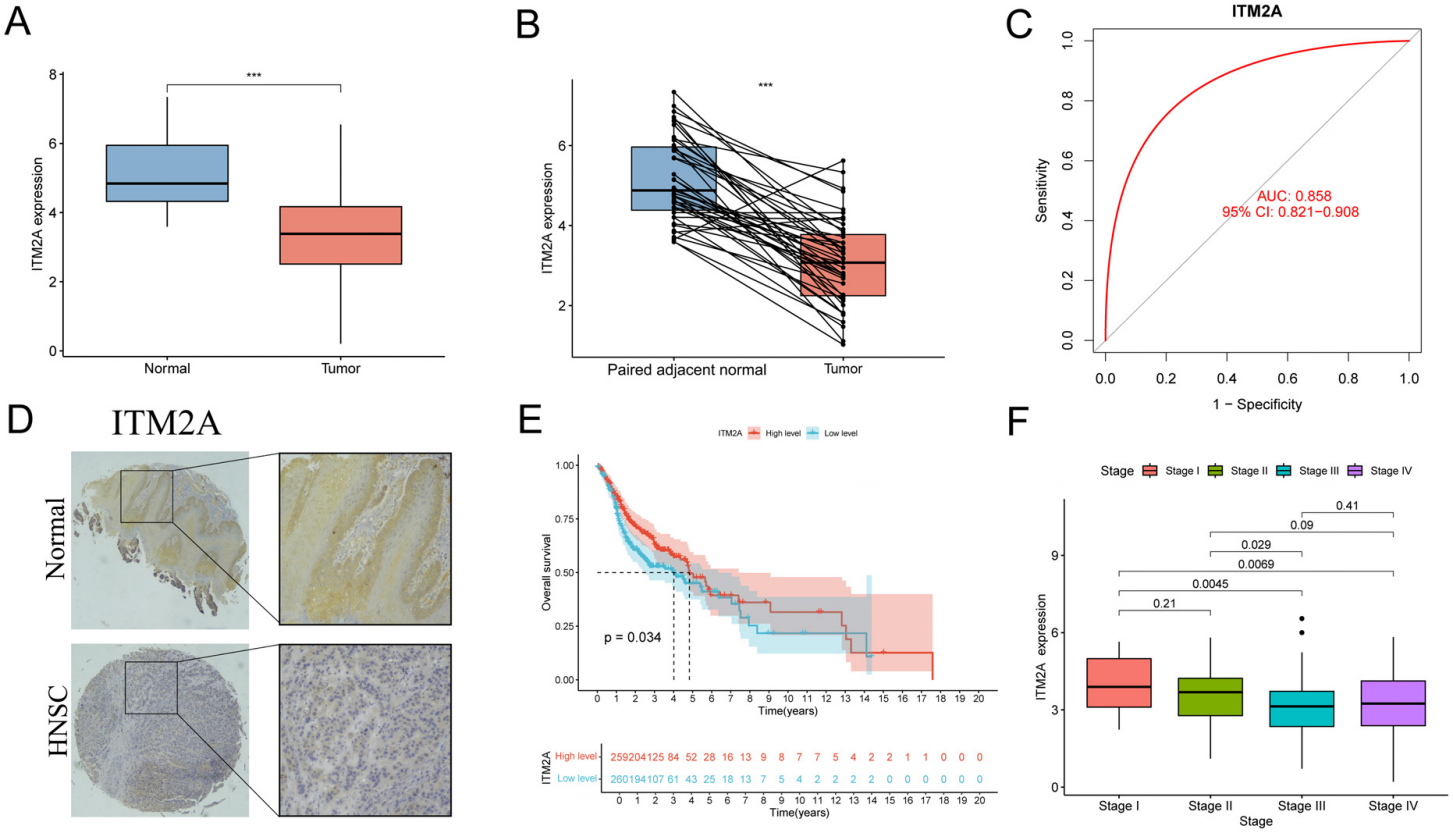
Expression level of ITM2A in HNSC and its clinical significance. **(A)** Expression levels of ITM2A in normal and HNSC tumor tissues. **(B)** Expression levels of ITM2A in paired normal and HNSC tumor tissues. **(C)** ROC curves of ITM2A expression for identification of HNSC tissues. **(D)** Representative image of ITM2A immunohistochemistry. **(E)** Kaplan-Meier curves of OS. **(F)** Tumors in different clinical stages (stages I-IV) Comparison of ITM2A expression levels. ***p<0.001.

We performed an in-depth analysis of tumor tissue (n=522) and normal tissue data (n=44) from the TCGA-HNSC cohort. ITM2A mRNA was significantly reduced in tumor tissues (**Figures 4A, B**). To further elucidate the diagnostic value of ITM2A in HNSC, we visualized it by ROC curve (**Figure 4C**). The AUC of ITM2A was 0.858 (95% CI: 0.821-0.908), which indicated the good efficacy of ITM2A in the diagnosis and prediction of HNSC. Immunohistochemistry experiments further supported this finding by demonstrating that the protein expression of ITM2A was also reduced in tumor tissues (**Figure 4D**; **Supplementary Figure S1**). Using Kaplan-Meier survival curves, low expression levels of ITM2A were associated with poorer overall survival (OS) in HNSC patients (**Figure 4E**, p=0.034). In addition, we found that the expression of ITM2A was significantly higher in early stage across different disease stages (**Figure 4F**).

### Potential functions of ITM2A in HNSC

**Figure 5:**
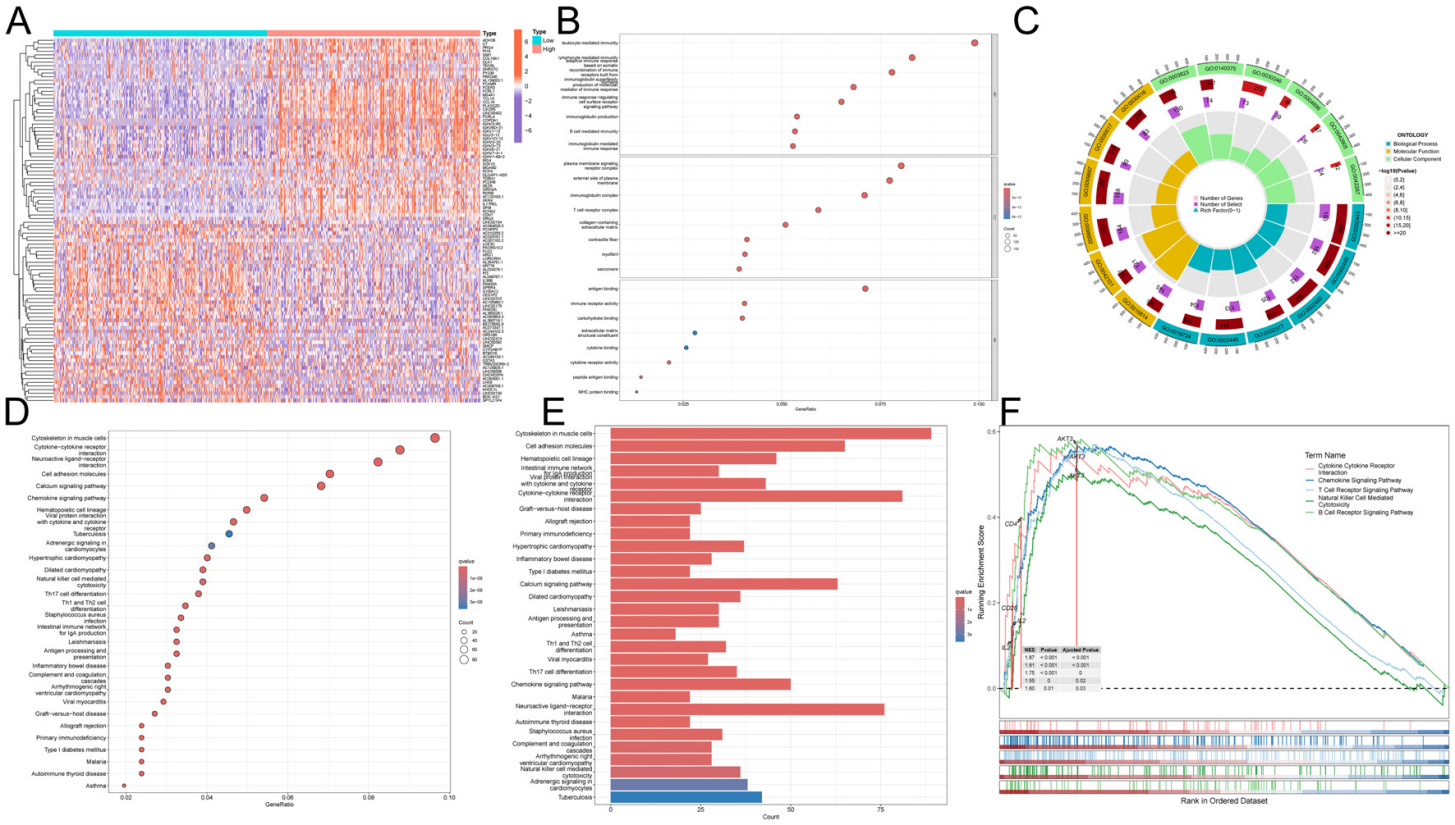
Functional role of ITM2A in HNSC. **(A)** Heatmap showing DEGs associated with ITM2A expression. **(B, C)** GO enrichment analysis. **(D, E)** KEGG enrichment analysis. **(F)** GSEA enrichment analysis of KEGG.

We demonstrated the top 50 DEGs correlated with ITM2A by heatmap clustering (**Figure 5A**). ITM2A-associated DEGs were enriched in T cell receptor complex, lymphocyte mediated immunity, production of molecular mediator of immune response, immune receptor activity and B cell mediated immunity by GO analyze (**Figures 5B, C**). Meanwhile, the results of KEGG enrichment analysis suggested that these DEGs might be involved in signaling pathways such as Primary immunodeficiency, Th1 and Th2 cell differentiation and Natural killer cell mediated cytotoxicity (**Figures 5D, E**). We also performed enrichment analysis of the KEGG reference gene set using GSEA, and found that enrichment in immune cell signaling pathway (**Figure 5F**). These results suggest that ITM2A and its related genes may play an important regulatory role during HNSC immune response.

### ITM2A is associated with tumor immune infiltration in HNSC

**Figure 6:**
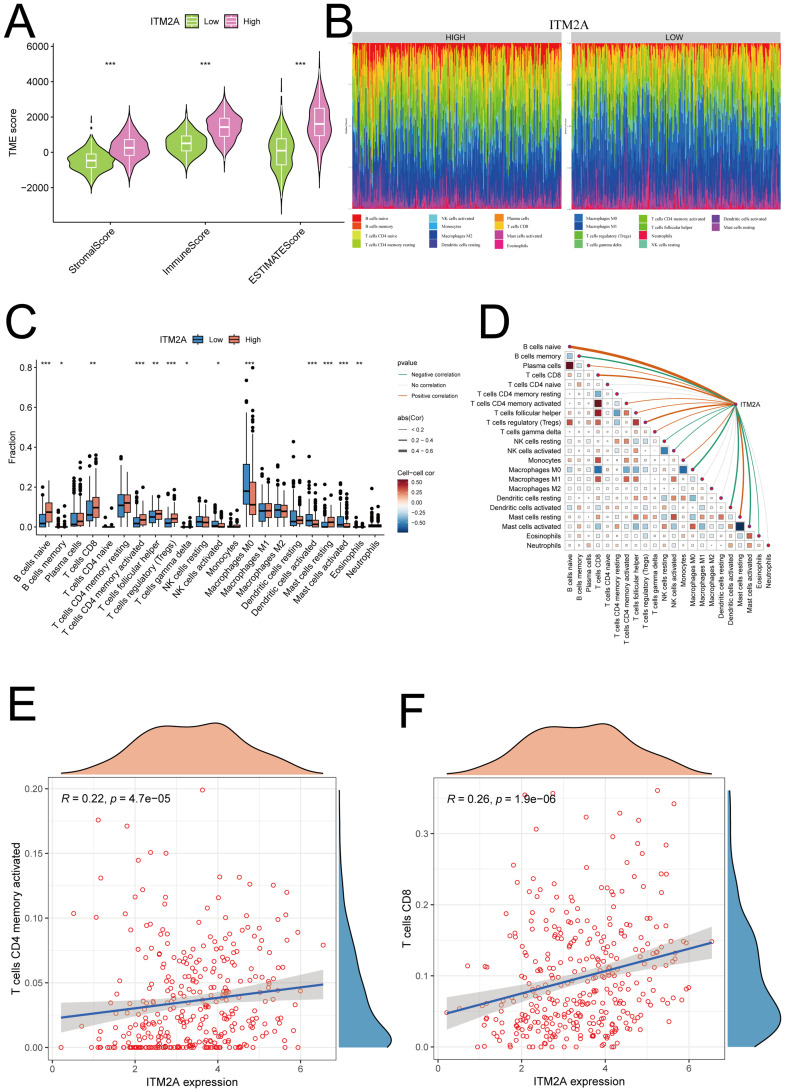
Relationship between ITM2A and tumor immune microenvironment. **(A)** Significant correlation of ITM2A with ImmuneScore, StromalScore and ESTIMATEScore. **(B)** Relative proportions of different immune cell types in HNSC samples. **(C)** Distribution of different types of immune cells in high and low ITM2A subgroups. **(D)** Correlation analysis of the level of infiltration of ITM2A and each type of immune cells. **(E)** Correlation analysis of ITM2A and CD4+ memory T cells. **(F)** Correlation analysis of ITM2A and CD8+ T cells. *p<0.05, **p<0.01, ***p<0.001.

Since the composition of TIME has an important impact on tumor development and subsequent treatment, we performed TIME scoring on TCGA-HNSC samples and analyzed its correlation with ITM2A expression. The results showed that ITM2A low patients had less immune and stromal cells (**Figure 6A**). This suggests that low ITM2A expression may be associated with a less active immune response, which may result in a decreased immune cell infiltration at the tumor site, such as reduced numbers and activities of T cells, B cells, and macrophages. To verify this speculation, CIBERSORT algorithm was used to calculated the immune cell infiltration (**Figure 6B**). It was found that the proportions of B cells naive, T cells CD8, T cells CD4 memory activated, T cells regulatory (Tregs) and Mast cells resting were increased in the samples with high ITM2A expression (**Figure 6C**). Meanwhile, ITM2A was positively correlated with B cells naive, T cells CD8, T cells CD4 memory activated, Tregs, and Mast cells resting, but negatively correlated with B cells memory, NK cells resting, and Mast cells activated, etc. (**Figures 6D–F**). This suggests that ITM2A appears to have a complex and selective regulatory effect on different types of immune cells, which may affect immune escape and response to immunotherapeutics.

### ITM2A may be a predictive marker for immunotherapy

**Figure 7:**
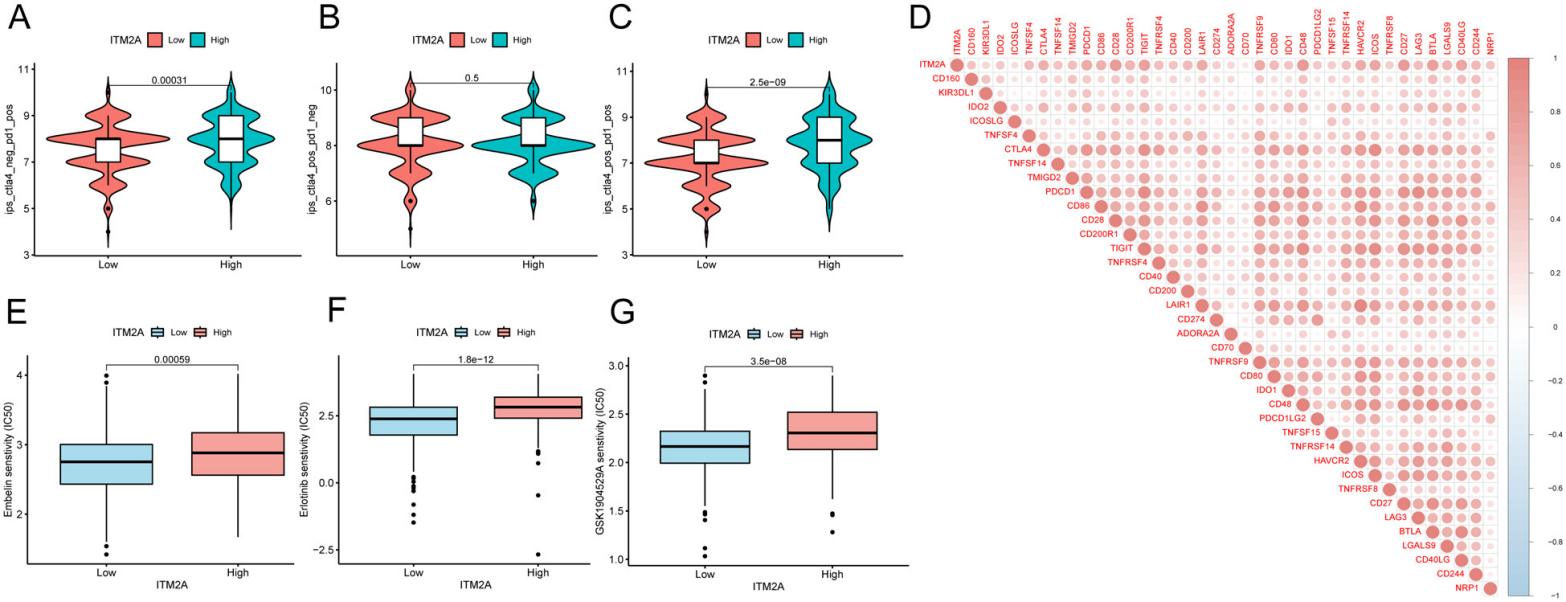
Correlation between ITM2A expression and immunotherapy and chemotherapy in HNSC. **(A–C)** Violin plots demonstrating whether there is a difference between HNSC patients in the high and low ITM2A expression groups in response to treatment with PD-1 and or CTLA_4 inhibitors. **(D)** Association between ITM2A expression and immune checkpoints. **(E–G)** Semi-inhibitory concentration values of all three chemotherapeutic agents were elevated in patients with high ITM2A expression in HNSC.

Analysis of ICP showed that samples with ITM2A(low) group had a reduced IPS in the case of PD1 ICP inhibitor treatment (**Figure 7A**). There is no difference of IPS in the in the case of ALT4 ICP inhibitor treatment (**Figure 7B**). However, ITM2A(low) group had a lower response to PD1+ALT4 ICP inhibitor therapy (**Figure 7C**). Immune checkpoint analysis showed that ITM2A is associated with immune checkpoints (**Figure 7D**). These findings further support that patients with low ITM2A have reduced responses to immunotherapy. Moreover, Embelin, Erlotinib, and GSK1904529A’s IC50 values were relatively lower in the low ITM2A expression group, suggesting that they were more sensitive to these drugs (**Figures 7E–G**). Together, these findings suggest that ITM2A is not only valuable in immunotherapeutic response, but may also have an impact on patient-specific drug sensitivity.

### Single-cell analysis of ITM2A in tumor immune cells

**Figure 8:**
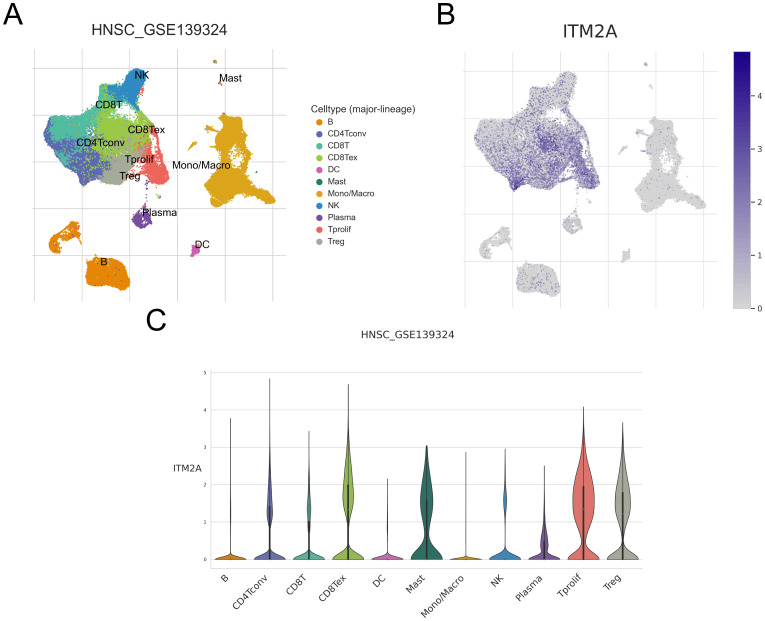
Expression of ITM2A in different cell types of HNSC in single cells. **(A)** UMAP plots showing the distribution of different clusters as well as annotations. **(B, C)** Distribution of ITM2A in different cell types.

We analyzed the GSE139324 dataset using the single-cell database TISCH. We successfully identified 23 different cell clusters, and further based on the marker gene expression patterns, we classified these cell clusters into one of 11 different cell types, including B cell, CD4Tconv, CD8T, CD8Tex, DC, Mast, Mono/Macro, NK cell, Plasma, Tprolif and Treg (**Figure 8A**). We found that ITM2A was expressed at higher levels in CD4Tconv, CD8T, CD8Tex, Mast, Tprolif, and Treg, and at lower levels in B cells, DC, Mono/Macro cells, and Plasma (**Figures 8B, C**). We also showed ITM2A expression in these cells based on TNM stage or Source (PBMC, TIL and Tonsil) in **Supplementary Figure S2**. Moreover, we used GSE10332 dataset to show ITM2A expression pattern across different cell types (**Supplementary Figure S3**). These results suggest that ITM2A plays a key role in the TIME of HNSC.

### Upregulation of ITM2A inhibits HNSC cell growth

**Figure 9:**
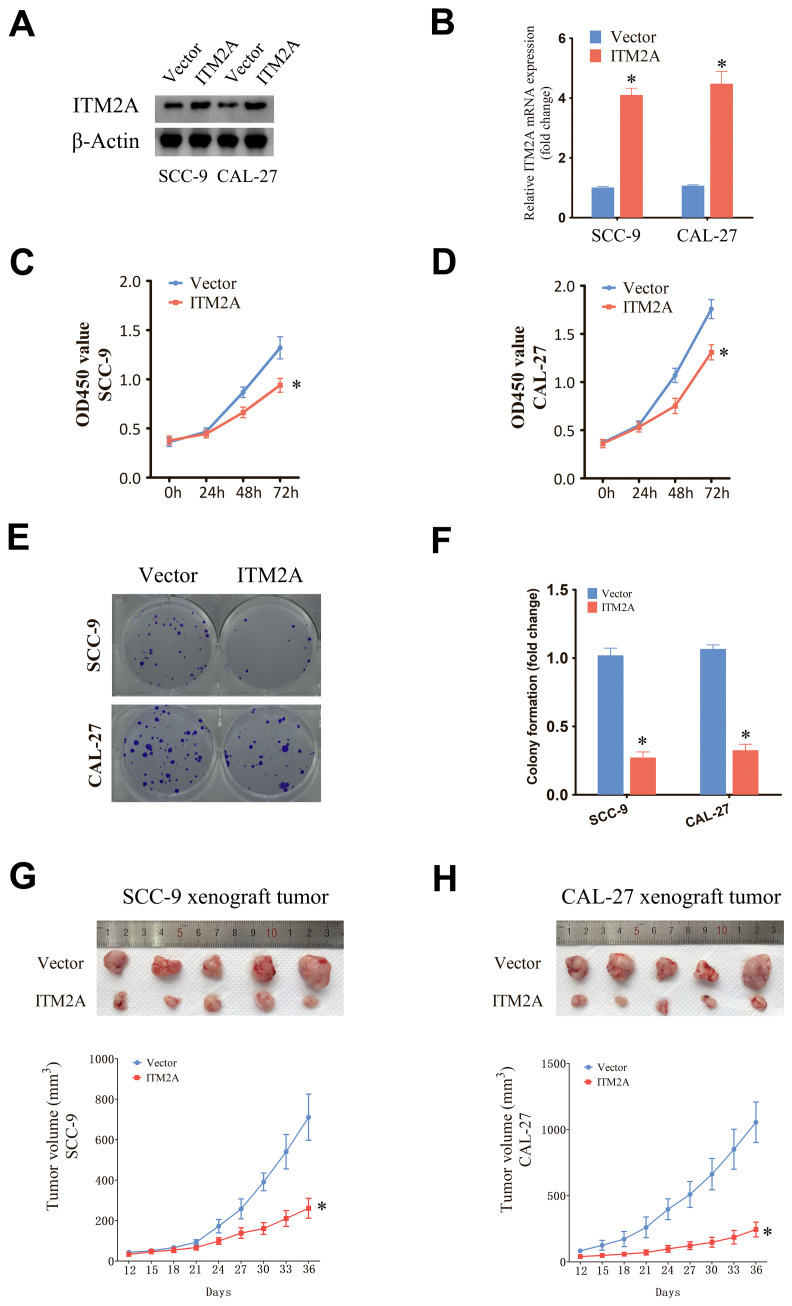
Upregulation of ITM2A inhibited the proliferation of HNSC cells. **(A, B)** Protein and mRNA levels were detected after transfection of empty vector or OE-ITM2A. **(C, D)** CCK-8 assay showing the proliferative capacity of HNSC cell lines with ITM2A overexpression. **(E, F)** Clone formation assay showing the clone formation ability of HNSC cell lines with ITM2A overexpression. **(G, H)** Xenograft tumor models were established using empty vector or ITM2A overexpression HNSC cells and tumor volumes were measured. The data represent the mean ± SEM. *p<0.05, versus vector.

Overexpressed ITM2A was determined by protein and mRNA level in SCC9 and CAL27 cells (**Figures 9A, B**). Subsequently, we evaluated the proliferation of SCC9 and CAL27 cells with ITM2A overexpression by CCK-8 assay (**Figures 9C, D**). The findings indicated that ITM2A overexpression markedly suppressed the OD450 value of SCC9 and CAL27 cells. Furthermore, ITM2A overexpression also diminished the clonogenic potential of SCC9 and CAL27 cells (**Figures 9E, F**). To further confirm *in vitro* results, we used SCC9 and CAL27 cells overexpressing ITM2A to construct xenograft model *in vivo*. Compared with the vector group, the tumor volumes of the ITM2A overexpression were significantly decreased (**Figures 9G, H**). In summary, ITM2A plays an anti-oncogene role in HNSC.

## Discussion

The pathogenesis of HNSC is multifactorial, encompassing smoking, alcohol consumption, viral infections, genetic predispositions, and environmental exposures, all of which are recognized as significant risk factors (1, 2). Current therapeutic strategies for HNSC primarily involve a multimodal approach, integrating surgical intervention, radiation therapy, chemotherapy, and immunotherapy. However, the treatment of intermediate and advanced patients still face major challenges.

TIME contains tumor cells, fibroblasts, diverse immune cells, various cytokines, extracellular matrix and endothelial cells. This milieu is pivotal in the context of tumor immunotherapy (26, 27). In HNSC, TIME has been applied to the screening of prognostic-related target targets (28). TIME-related genes are instrumental in evaluating patient prognosis and therapeutic efficacy. Consequently, further investigation into TIME-related genes is imperative. Machine learning, a technology that employs algorithms and extensive datasets for pattern recognition and prediction, can analyze vast databases, uncover latent patterns and relationships, and autonomously identify features and trends without dependence on *a priori* assumptions. This capability addresses the limitations of traditional screening methods by providing a multifaceted approach to target identification (29). In order to further screen the prognostically relevant DEGs, we used a one-way COX analysis and 20 prognostically relevant DEGs were identified. including: TRAV26-1, IGKV1-16, TRAV8-5, TRAV13-1, IGHV2-5, IGHV1-46, PLA2G2D, CD28, IGHV3-64, RSPO1, TRBV6-6, TRAV8-4, FOXP3, CCR8, TRAV2, TCL1A, SELL, WIPF1, and ITM2A. In order to identify which DEGs are the key prognostic genes associated with TIME, more in-depth screening and research is needed. We further screened these DEGs using various machine learning algorithms, including LASSO, Randomforst, SVM-RFE, and GBM, and identified four key genes: RSPO1, FOXP3, WIPF1, and ITM2A. Among them, RSPO1 was associated with the cancer phenotype of palmoplantar keratosis and showed metastasis-related features in colon cancer (30). FOXP3 was closely associated with a variety of tumors, and involved in tumor immunotherapy (31). WIPF1 regulated tumor development through the PI3K/AKT pathway and was involved in immune responses in gastric cancer (32). ITM2A is a protein-coding gene that belongs to the ITM2 family (8). ITM2A was associated with the process of iron apoptosis in hepatocellular carcinoma and involved in the regulation of the immune system (33). In cervical cancer, ITM2A can be used as a predictive marker for overall survival prognosis. However, the role of ITM2A in HNSC is not understood and requires further research.

By bioinformatics analysis, ITM2A expression was significant reduced and associated with immune cells in most tumors. ITM2A mRNA levels were enhanced in normal tissues and this finding was verified by IHC experiments. We also found that high ITM2A expression group have a better overall survival. By analyzing the clinical data, we found that ITM2A expression was higher in the early stages of the tumor, and its expression level began to decrease as the tumor stage progressed. GO enrichment analysis showed that ITM2A-associated DEGs were enriched in immune response-regulating, immunoglobulin mediated, immune response, B cell mediated immunity. KEGG enrichment analysis showed that ITM2A was associated with receptor for IgA production, natural killer cell mediated cytotoxicity, Th1 and Th2 cell differentiation, Chemokine signaling pathway. To further explain the involvement of ITM2A in immune response in HNSC from multiple perspectives, we also performed enrichment analysis of KEGG using GSEA. The results showed that ITM2A was enriched into Cytokine Cytokine Receptor, Interaction, Chemokine Signaling Pathway, T Cell Receptor Signaling Pathway, Natural Killer Cell Mediated Cytotoxicity and B Cell Receptor Signaling Pathway, and is associated with AKT3, CD4, CD28 and IL2. These pathways are closely linked to the immune response (34, 35), which implies that ITM2A is involved in the immune response of tumors.

The TIME of HNSC was analyzed by using the ESTIMATE algorithm, and we observed that ImmuneScore, StromalScore and ESTIMATEScore were elevated in ITM2A high expression group. CIBERSORT algorithm showed that ITM2A expression was associated with immune cell proportions. More importantly, we found that ITM2A expression was correlated with CD4 memory activated and CD8 cells. In gastric cancer, T cell CD4 memory activated is involved in its prognosis through metabolic reprogramming, which directly leads to apoptosis and correlates with ferroptosis by releasing substances such as perforin and granzyme (36, 37). The close association of ITM2A with immunomodulation triggered an in-depth exploration of its impact in immunotherapy. We found that ITM2A expression had a significant effect on PD-1 and CTLA-4 inhibitors, especially in the clat4_neg_pd1_pos and clat4_pos_pd1_pos groups. Further analysis revealed that immune checkpoint genes such as CD160, KIR3DL1, IDO2, TNFSF4, TNFSF14, CTLA4, CD28, CD274, CD80, and NRP1 were related to ITM2A expression. KIR3DL1 could serve as a relevant marker for immunosurveillance in cervical cancer cells (38).The immune responses of IDO2 and B cells are closely related. Inhibitors of CTLA4 are used in the immunotherapy of numerous tumors (39). In breast cancer, CD80 and tumor cell efficacy in chemotherapy are strongly correlated (40). The correlation between ITM2A and these immune checkpoint genes further illustrates its potential role in the mechanisms of immune escape and immune surveillance in HNSC. We also investigated ITM2A in HNSC at the single-cell level. ITM2A was higher in CD4Tconv, CD8T, CD8Tex, Mast, Tprolif, and Treg. These immune cells are involved in immunosuppressive process (41, 42), thus ITM2A may affect the immune escape of HNSC. Notably, we found that Embelin, Erlotinib and GSK1904529A had better sensitivity when ITM2A was highly expressed. However, screening drug sensitivity based on expression has some limitations and more validation is needed before clinical application.

We explored the impact of ITM2A on HNSC through cell and animal experiments. Overexpression of ITM2A decreased cell proliferation and colony formation in HNSC cells. Additionally, the tumor volume in animal experiments was also significantly reduced. These findings indicate that elevated ITM2A expression inhibits HNSC growth. Our study suggests that ITM2A is a potential therapeutic marker for HNSC, closely associated with the TIME and prognosis, as demonstrated through bioinformatics analysis and experiments, there are still some limitations. The specific mechanism of ITM2A regulating immune infiltrating cells needs further experimental verification. In addition, the effect and mechanism of action of ITM2A on HNSC need further experimental exploration. Nevertheless, our study provides new insights and ideas for the study of HNSC in TIME-related aspects and the exploration of new therapeutic modalities.

## Conclusion

In summary, the expression of ITM2A was markedly diminished in the tissues of patients with HNSC, and this reduction was significantly correlated with adverse prognosis and tumor progression. Furthermore, ITM2A expression is intimately linked to TIME. The findings of this study indicate that ITM2A serves not only as a prognostic biomarker for predicting disease progression in HNSC but also potentially plays a crucial role in immune-related activities.

## Data Availability

The original contributions presented in the study are included in the article/**Supplementary Material**. Further inquiries can be directed to the corresponding authors.
