# Supplementary material for: Integrating machine learning, bioinformatics and experimental verification to identify a novel prognostic marker associated with tumor immune microenvironment in head and neck squamous carcinoma
Source: Front Immunol. 2024 Dec 10;15:1501486. doi: 10.3389/fimmu.2024.1501486 (PMC11666523; doi:10.3389/fimmu.2024.1501486)
Supplement: Supplementary file 1 [file DataSheet1.doc]

**Supplementary Material**


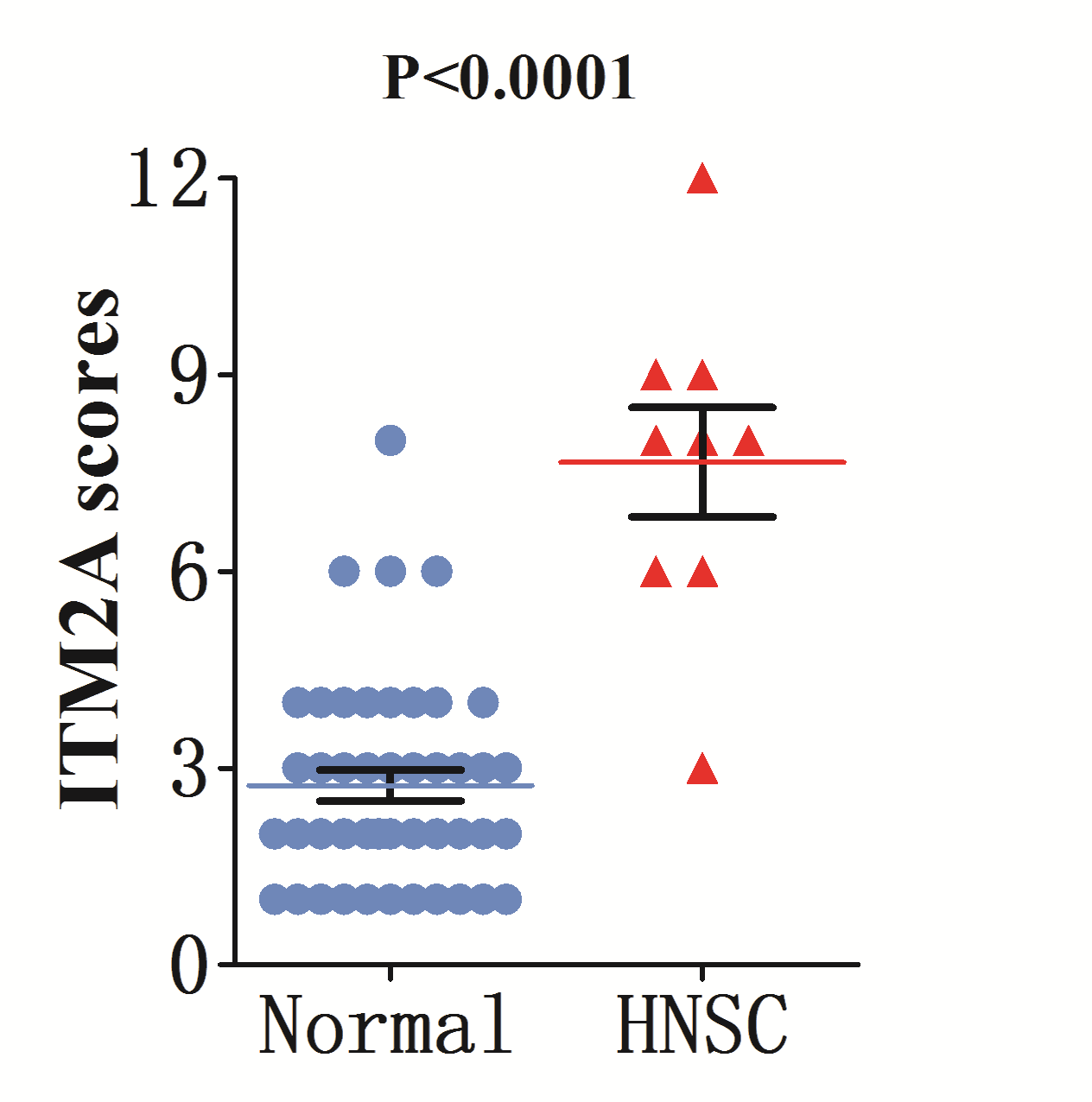


Supplementary Figure S1. Scores of ITM2A protein expression in normal tissues (n = 9) and HNSC tissues (n = 45).


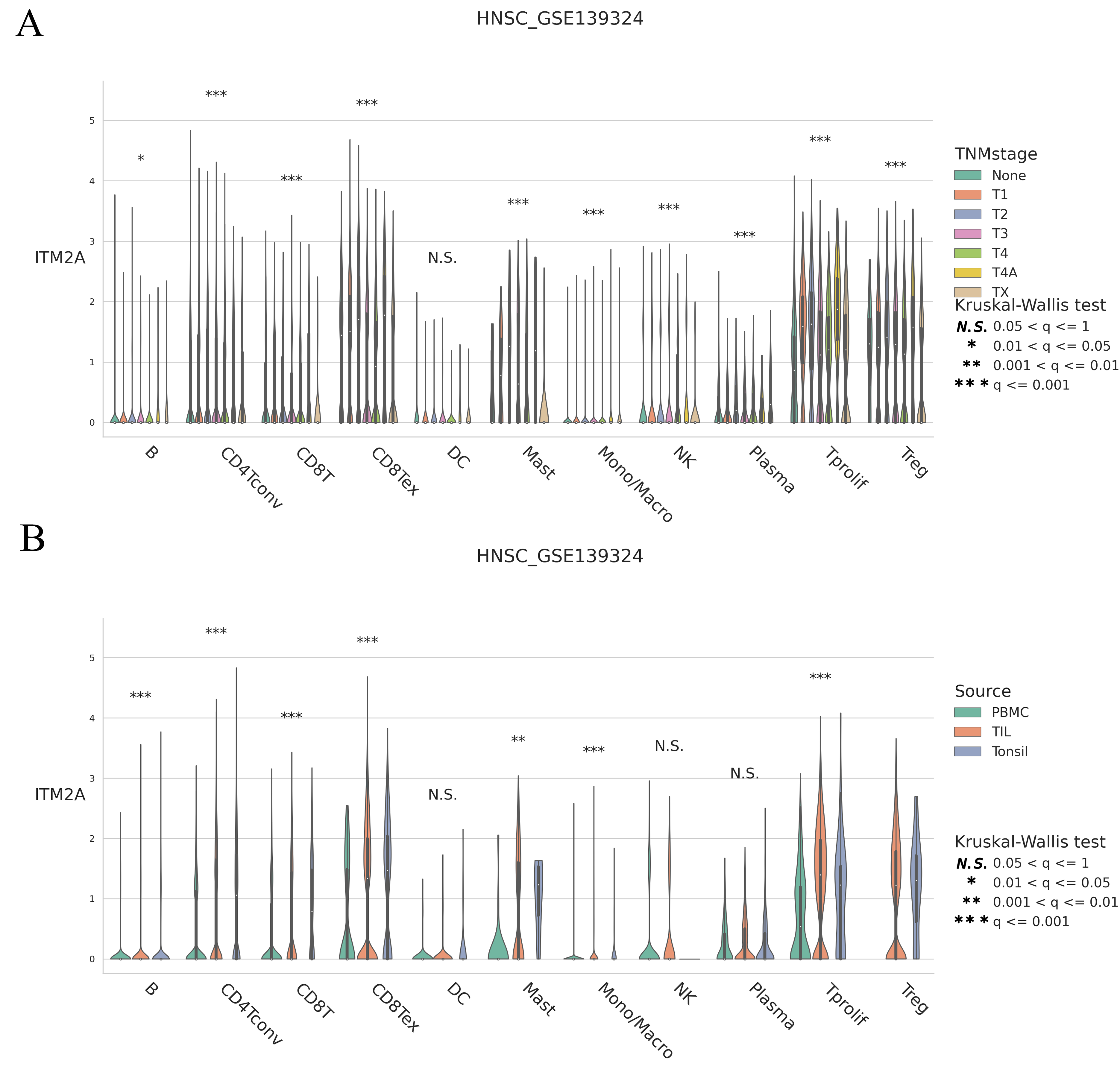


Supplementary Figure S2. A. ITM2A expression of TNM stage in immune cell subtypes. B. ITM2A expression of PBMC, TIL and Tonsil in immune cell subtypes.


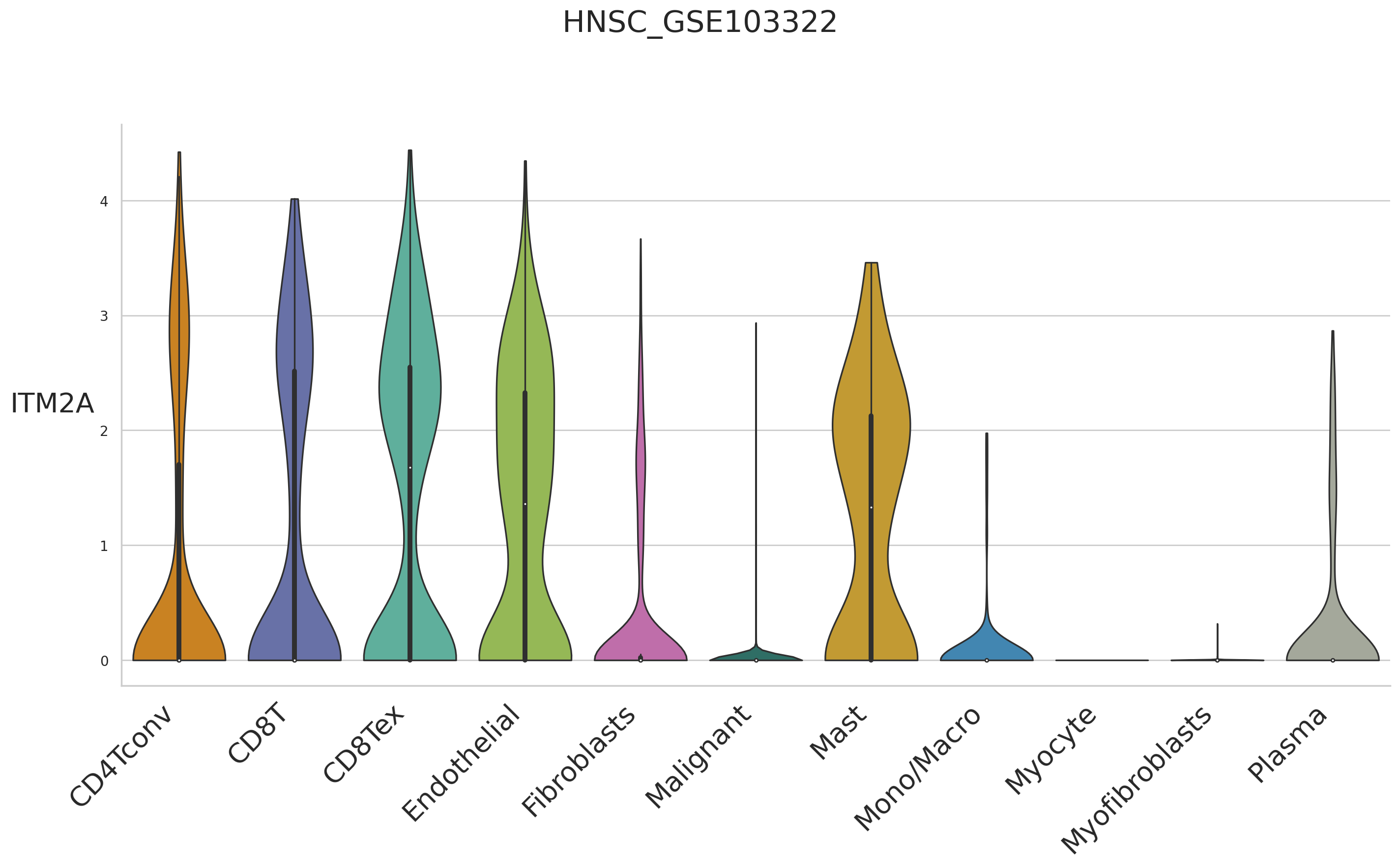


Supplementary Figure S3. Distribution of ITM2A in different cell types (GSE103322).
